# Sacrospinous ligament fixation: medium and long-term anatomical results, functional and quality of life results

**DOI:** 10.1186/s12905-021-01195-7

**Published:** 2021-02-12

**Authors:** Angeline Favre-Inhofer, Marie Carbonnel, Rouba Murtada, Aurélie Revaux, Jennifer Asmar, Jean-Marc Ayoubi

**Affiliations:** 1 Department of Obstetrics Gynecology and Reproductive Medecine, Hospital Foch, Suresnes, France; 2 University Versailles, Saint-Quentin en Yvelines, France

**Keywords:** Sacrospinous fixation, Anatomical results, Pelvic organ prolapse, Quality of life

## Abstract

**Background:**

To evaluate the medium and long-term anatomical results of sacrospinous ligament fixation (SLF) and its impact on quality of life (QoL).

**Methods:**

We conducted a retrospective and observational single centre study. Fifty-nine patients were interviewed using the Pelvic Floor Distress Inventory and Pelvic Floor Impact Questionnaire and underwent physical examination using POP-Q several years after SLF. Primary outcome was the comparison of anatomic results of SLF at medium-term (group 1: 1–5 years after surgery) and long-term (group 2: more than 5 years after surgery). The secondary outcome was QoL evaluation.

**Results:**

The overall recurrence and complication rates were respectively 22% and 10%, with no significant differences between groups 1 and 2. The recurrence rate was similar in both groups [twelve (35%) in group 1 and nine (20%) in group 2, *p* = 0.09]. Two patients (12%) in the recurrence and none in the no recurrence group had clinical symptoms (*p* = 0.08). Two patients (12%) in the recurrence and one patient (2%) in the no-recurrence group had a significant impact on their quality of life (*p* = 0.12).

**Conclusion:**

This study showed sustainable anatomic and functional results of SLF in medium and long-term analysis with overall low morbidity.

## Background

Pelvic organ prolapse (POP) is a common condition in elderly women. Although not a life-threatening condition, it affects quality of life. The prevalence of this disorder is between 2.9 and 11.4% when using questionnaires and 31.8 to 97.7% when using physical examination [1] with Pelvic Organ Prolapse Quantification System (POP-Q) [2]. This wide variation in prevalence rates is explained by the fact that authors may consider either POP-Q stages I or II as positive diagnostic of prolapse. Furthermore, stages may differ according to the method of diagnosis. When using questionnaires, women complaining of prolapse are more often found to have a stage II and more prolapse upon physical examination. It is estimated that eleven percent of women over 70 years will undergo surgical treatment for pelvic organ prolapse [3]. The current gold standard for the diagnosis of POP is clinical examination using the POP-Q staging system, with prolapse defined as stage II and more.

Transvaginal surgical treatment is mainly represented by the sacrospinous ligament fixation (SLF) [4]. This surgical technique described in 1968 by Richter [5] consists in stitching the posterior vaginal wall to the sacro-spinous ligament. Compared to sacrocolpopexy, sacrospinous ligament fixation has a higher rate of dyspareunia and recurrence [6] with lower morbidity, shorter intervention time and faster postoperative recovery [7]. It also has a lower cost [8].

Few studies have analysed the long-term anatomic results although it is an essential aspect of prolapse surgery. The recurrence rates in literature vary widely, ranging from 0 [9] to 70.3% in the OPTIMAL trial [10]. This recent randomised clinical trial compared uterosacral ligament suspension versus SLF, probably using overly strict recurrence criteria. The recurrence rates were respectively 61.5% and 70% without any significant difference.

There are various symptoms associated with pelvic organ prolapse [1]: urinary symptoms (stress, urge or mixed urinary incontinence), bulge symptoms (heaviness) and colo-rectal symptoms (constipation and dyschezia) [11].

Quality-of-life is a crucial element when studying functional surgeries. The Pelvic Floor Distress Inventory (PFDI-20) questionnaire [12], validated for French language, evaluates prolapse symptoms and includes prolapse, ano-colo-rectal and urinary questions. The Pelvic Floor Impact Questionnaire short form (PFIQ-7) evaluates the urinary, prolapse and ano-colo-rectal consequences of the prolapse in daily activities. These two questionnaires allowed us to evaluate quality of life in prolapse patients.

Our primary objective was to evaluate the medium and long-term anatomical results of SLF. Our secondary objective was to evaluate its impact on the quality of life.

## Methods

This study was a retrospective and observational single centre study. We contacted all the patients who underwent SLF between January 1, 2008 and December 31, 2018 at Foch hospital (Suresnes, France). If the patient agreed to enter the study, an information letter was sent. Medical and surgical history, initial purpose of consultation, urinary and anorectal symptoms, physical examination using the POP-Q system [2] to assess prolapse stage, perioperative data, hospitalisation data, postoperative data and recurrence or the need for secondary urinary incontinence surgery were collected retrospectively from medical files. To assess medium and long-term anatomical results patients underwent a clinical examination using POP-Q prolapse stage and measuring the different POP-Q points and a detailed interview with quality-of-life questionnaires between January and April 2019. Presence of symptoms associated with POP such as urinary symptoms (stress urinary incontinence, overactive bladder symptoms), sexual symptoms (dyspareunia), bulge symptoms (heaviness, bulge) and ano-rectal symptoms (dyschezia, fecal incontinence) was searched in medical files for the pre-operative and early post-operative period, and evaluated by the clinician through questionnaires and interrogation during the interview. Physical examination was performed by one clinician. Prolapse recurrence was defined as anatomic prolapse recurrence for a POP-Q > 1 or repeat surgery for prolapse. Urinary incontinence was defined as urine leakage after a cough test. Medium and long-term were defined as 1–5 years following surgery (group 1) and 5–10 years (group 2), respectively. Two quality-of-life questionnaires were used. The first one was the PDFI-20, a 20-item questionnaire, validated in French [12] and separated into three subscales. The Urinary Distress Inventory 6 (UDI-6) explores the urinary symptoms of prolapse, the Colorectal-Anal Distress Inventory 8 (CRADI-8) colorectal symptoms and finally the Pelvic Organ Prolapse Distress Inventory 6 (POPDI-6) bulge and heaviness symptoms. The second quality-of-life questionnaire used was the Pelvic Floor Impact Questionnaire short form (PFIQ-7) [12], which appreciates the impact of urinary, colo-rectal and prolapse symptoms on quality of life. Defining satisfactory or unsatisfactory scores is difficult for both questionnaires. A PFDI-20 score higher than 62/300 defines a symptomatic (bothersome) prolapse, as mentioned by Letouzey et al. [13]. A PFIQ-7 score higher than 100/300 was defined as impacting quality of life.

Chi2 and Fisher tests were used for categorical variables and T student or Mann–Whitney tests were used for average comparisons. SAS® software (V. 9.4) was used for the analysis. Statistical tests were 2-sided and significance was evaluated at an α level of 0.05.

Our trial was approved by the French Authority, the Advisory Committee on Information Processing in Healthcare Research (ID-CRB: 2018-218 C25). This protocol obtained the agreement of the National Commission for Data Protection and Liberties (CNIL-France) by respecting the reference methodology.

### Licensing information

The Pelvic Organ Prolapse Quantification System (POP–Q) is distributed under the terms of the Creative Commons Attribution License, which permits unrestricted use, distribution, and reproduction in any medium, provided the original work is properly cited.

The Pelvic Floor Distress Inventory and the Pelvic Floor Impact Questionnaire are licensed under the terms of the Creative Commons license.

## Results

**Fig. 1:**
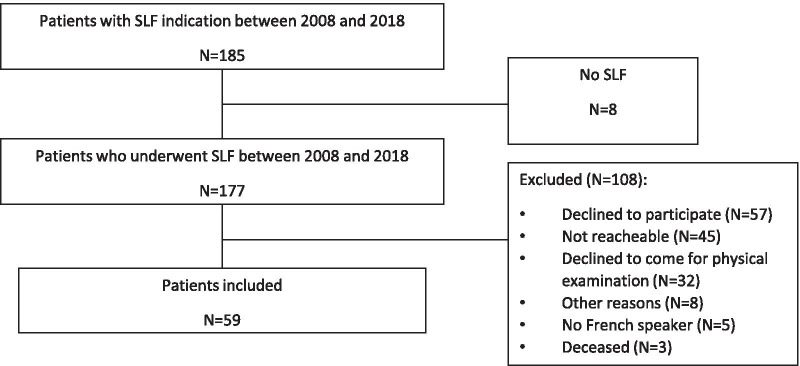
Flow chart database searching pathway and group divisions

A total of 185 patients were referred for SLF over the study period. 177 patients eventually underwent surgery (Fig. 1) and 108 were excluded: 57 declined participation, 45 were not reachable, 32 declined to come in for examination, 5 were not fluent French speaker, 3 were deceased (unrelated to the surgery) and 8 were not included for other reasons (mostly because of dementia).

59 patients were interrogated and underwent physical examination. Patient demographics, past medical history*,* pre-operative symptoms, intra-operative data, and short-term complications are presented in Table 1. Mean age at surgery was 63.6 years. Fifty-three patients (90%) had surgical history, 50 (85%) were post-menopausal and 53 (90%) were multiparous. Thirty-four patients were interviewed and examined 5 years or less after surgery (Group 1) and 25 patients more than 5 years after surgery (Group 2).

**Table 1:** Demographic data, past medical history, pre-operative symptoms, intra-operative data and short-term complications for overall population, group 1 and group 2

	Overall population (n = 59)	Group 1 (n = 34)	Group 2 (n = 25)	*p*
*Socio-demographic data and previous history*				
Age at surgery (years) [mean (SD)]	63.6 (± 8.0)	62.8 (± 8.7)	63.6 (± 7.8)	0.37
Surgical history	53 (90%)	30 (88%)	23 (92%)	0.32
History of abdominal surgery	29 (49%)	18 (54%)	18 (72%)	0.08
History of hysterectomy	12 (20%)	6 (18%)	6 (24%)	0.28
Menopause	50 (85%)	29 (52%)	21 (84%)	0.44
*Parity*				
Nulliparity	0 (0%)	0 (0%)	0 (0%)	
Primiparity	6 (10%)	3 (9%)	3 (12%)	0.19
Multiparity	53 (90%)	31 (91%)	22 (88%)	
*Preoperative symptoms*				
Stress urinary incontinence	16 (27%)	13 (38%)	3 (12%)	0.01*
Urge urinary incontinence	19 (32%)	10 (29%)	9 (36%)	0.3
Dyspareunia	3 (5%)	2 (6%)	1 (4%)	0.35
*Anesthesia*				
General anaesthesia	56 (97%)	34 (100%)	23 (92%)	0.08
Spinal anaesthesia	2 (4%)	0 (0%)	2 (8%)	
*Concomittant surgery*				
Total hysterectomy	47 (79%)	29 (85%)	18 (25%)	0.12
Anterior compartment surgery	36 (61%)	25 (73%)	11 (44%)	0.01*
Posterior compartment surgery	43 (73%)	25 (73%)	18 (72%)	0.45
Postoperative complications	6 (10%)	3 (9%)	3 (12%)	0.35

Group 1: ≤ 5 years after SLF; group 2: > 5 years after SLF. Chi2 and Fisher tests were used for categorical variables and T student or Mann–Whitney tests were used for average comparisons

SD, standard deviation

*: significant result

Fifty-six (97%) patients underwent SLF under general anaesthesia (Table 1). As for concomitant procedures, 47 patients (80%) had a total hysterectomy as an additional procedure, 36 (61%) had an anterior vaginal wall reconstruction (in 31 colporrhaphy and in 5 anterior mesh surgery) and 43 (73%) had a posterior vaginal wall surgery (posterior colporrhaphy). Six patients (10%) had postoperative complications: two urinary retentions, two wound infections, one urinary tract infection, one haematoma. Table 2 summarizes recurrence, mid and long-term complications. Upon physical examination at time of study, 32 (54%) patients had stress urinary incontinence and 13 (22%) had prolapse recurrence. When considering the 19 (32%) patients who were still sexually active, eight (42%) had dyspareunia. Prolapse recurrence occurred in a total of 17 patients (29%). The mean PFDI-20 and PFIQ-21 scores were respectively 22 (± 17) and 15 (± 33) reflecting a good quality of life.

**Table 2:** Mid and long-term data for overall population, group 1 and group 2

	Overall group (n = 59)	Group 1 (n = 34)	Group 2 (n = 25)	*p*
Mediane of follow up (years) [min; max]	5 (1; 10)	3 (1; 5)	7 (6; 10)	NA
Age at exam (years) [mean (SD)]	69 (± 9)	66 (± 9)	71 (± 8)	0.01*
Urinary incontinence	32 (54%)	18 (53%)	14 (56%)	0.41
Fecal incontinence	2 (3%)	1 (3%)	1 (4%)	0.42
Sexual activity	19 (32%)	10 (29%)	9 (36%)	0.30
Dyspareunia	8 (42%)	4 (40%)	4 (44%)	0.32
Anatomic recurrence	13 (22%)	9 (26%)	4 (16%)	0.16
Anterior vaginal wall prolapse	13 (22%)	9 (26%)	4 (16%)	0.16
Apical vaginal prolapse	1 (2%)	1 (3%)	0 (0%)	0.16
Posterior vaginal wall prolapse	1 (2%)	1 (3%)	0 (0%)	0.16
Reintervention	4 (7%)	3 (9%)	1 (4%)	0.22
Overall recurrence	17 (29%)	12 (35%)	5 (20%)	0.09

Group 1: ≤ 5 years after SLF; group 2: > 5 years after SLF. Min: minimum time of follow up, max: maximum time of follow up, NA: not applicable. Chi2 and Fisher tests were used for categorical variables and T student or Mann–Whitney tests were used for average comparisons

SD, standard deviation

*: significant result

**Fig. 2:**
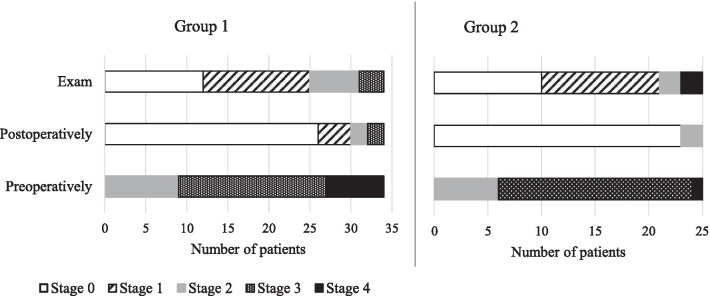
Prolapse stage for patients undergoing SLF in group 1 and group 2. Group 1: patients undergoing SLF ≤ 5 years; Group 2: patients undergoing SLF > 5 years ago. White content is stage 0, striped content is stage 1, grey content is stage 2, dotted content is stage 3 and black content is stage 4. Postoperatively: 1 month after surgery. SLF: sacrospinous ligament fixation

Figure 2 shows the distribution of prolapse stages in the two patient groups (1 to 5 years after surgery group and more than 5 years after surgery group). Table 1 compares the 2 populations and Table 2 details recurrence cases and complications.

Patient characteristics were statistically comparable between the two groups (Table 1). Median follow up was 3 years for group 1 and 7 years for group 2 (Table 2). The procedures were mainly performed by three experienced surgeons. There were more patients with preoperative stress urinary incontinence (13 versus 3, *p* < 0.01) and more anterior compartment procedures (25 versus 11, *p* < 0.01) in group 1. Not surprisingly, mean age at the time of the study was higher in group 2. The two groups were also comparable regarding the anatomic results shown in Fig. 2 and Table 2. Figure 2 illustrates the overall prolapse stage prior to surgery, one month post-operatively and at the time of study. In both groups, most prolapse cases were stage 3 preoperatively. At one month post-operatively, the recurrence rate was low in both groups (four (12%) in group 1 and two (8%) in group 2, *p* = 0.13) and increased at the time of study, with nine patients (26%) in Group 1 and four (16%) in group 2, (*p* = 0.16). Prolapse recurrence occurred mostly in the anterior vaginal wall. Three (9%) patients in group 1 and one (4%) patient in group 2 underwent surgery for recurrence (*p* = 0.22). The overall recurrence rate was also similar in both groups (12 patients (35%) in group 1 and 9 (20%) in group 2, *p* = 0.09).

Table 3 presents the mean scores for the PFDI-20 and PFIQ-21 questionnaires. There was no significant difference in PFDI-20 scores between groups 1 and 2 with a mean score of 25 (± 20) and 18 (± 13) (*p* = 0.06), respectively. Symptoms of prolapse defined by the POPDI-6 were significantly decreased in group 1 (7 (± 9)) compared to group 2 (3 (± 4), *p* = 0.02). Group 2 had a better quality of life than group 1 when considering PFIQ-21 and its sub-categories of prolapse (POPIQ-7) and urinary symptoms (UIQ-7). When considering the groups with or without recurrence, means for PFDI-20 and PFIQ-21 were similar. Means for PFDI-20 were 19 (± 13) for the no recurrence group and 17 (± 13) for the recurrence group (*p* = 0.32) while means for PFIQ-21 were 16 (± 27) for the no recurrence group and 15 (± 45) for the recurrence group (*p* = 0.46). Most of the patients in these two groups were asymptomatic or not bothered by prolapse symptoms and had a good quality of life, as confirmed by PFDI-20 and PFIQ-7. Two patients (12%) in the recurrence group had bothersome symptoms with a PFDI score greater than 62 (*p* = 0.08). No patient in the non-recurrence group complained of bothersome symptoms. Two patients (12%) in the recurrence group and one patient (2%) in the no-recurrence group had an impaired quality of life (*p* = 0.12).

**Table 3:** Mean of PFDI-20 and PFIQ-20

Quality of life [mean (SD)]	Total (n = 59)	Group 1 (n = 35)	Group 2 (n = 24)	*p*	No recurrence (n = 42)	Recurrence (n = 17)	*p*
PFDI-20	22 (17)	25 (20)	18 (13)	0.06	19 (13)	17 (13)	0.32
POPDI-6	5 (8)	7 (9)	3 (4)	0.02*	7 (5)	6 (7)	0.42
CRADI-8	6 (6)	7 (7)	5 (5)	0.14	9 (8)	8 (6)	0.32
UDI-6	11 (10)	11 (10)	10 (9)	0.3	3 (4)	3 (4)	0.35
PFIQ-21	15 (33)	22 (40)	6 (15)	0.02*	16 (27)	15 (45)	0.46
POPIQ-7	2 (8)	4 (10)	0 (0)	0.02*	2 (5)	3 (13)	0.46
CRAIQ-7	3 (10)	3 (11)	2 (10)	0.45	3 (27)	3 (10)	0.46
UIQ-7	11 (23)	16 (27)	3 (11)	0.01*	11 (22)	9 (24)	0.37

Group 1: ≤ 5 years after SLF; group 2: > 5 years after SLF. T student tests were used in this table

SD, standard deviation

*: significant result

## Discussion

This study demonstrates that SLF is an efficient long-term corrective surgery for uterine prolapse. Aside from early prolapse recurrences, few other cases occur. This is illustrated by the absence of significant difference in prolapse recurrence between patients operated more and less than 5 years prior to the study. Symptom scores and quality of life scores were high, and no difference in these parameters was noted when comparing patients who had a prolapse recurrence to those who did not.

The strength of our study is the long-time span of observation with a median follow up of 5 years. We used validated tools, with the POP-Q system to evaluate prolapse, as recommended by most medical associations [14] and PFDI-20/PFIQ-7 surveys, for which the French version has been validated [15]. The main weakness of our study is the fact that it was observational and retrospective, leading to information bias. There was a selection bias resulting from patients lost to follow up and an evaluation bias because the physical examination at time of study was performed by one single clinician. We were also unable to compare the different POP-Q points but only the POP-Q stage because detailed pre- and post-operative POP-Q points had not been reported in medical charts. Another bias is that even if the two groups (group 1 and group 2) have similar characteristics, they are not totally identical. A longitudinal study evaluating the less than 5 years after and more than 5 years after results in the same population would have been a good approach. Unfortunately, this has not been possible in our retrospective cohort.

When comparing to the cohorts reported in the literature, our population was equivalent in terms of age, menopausal status and preoperative prolapse stage (16, 17). Our 10% complication rate was also in accordance with those reported in the literature [16]. Our 5% severe complication rate (two wound infections and one haematoma) matches the literature rate (5.6%) represented by bladder or rectum injuries, anaesthesia complications, transfusion, wound infections, and rare death occurrences. This low morbidity rate makes SLF generally suitable for all women with prolapse including old and comorbid patients.

Long-term studies evaluating anatomic results more than 5 years after surgery are rare, with most studies limited to three-year observation periods. Paraiso et al. [18] found that 42% of the patients undergoing SLF had a recurrence. The recurrence-free rates at 1, 5 and 10 years were analysed and were respectively of 88.3%, 79.7% and 51.9%. The recurrence rates found were higher than ours. For Colombo et al.[19], Aksakal et al*.*[20] and Jelovsek et al. [10] recurrence rates were respectively of 27%, 25,7% and 27%. These rates were closer to those found in our study. Our 7% reintervention rate was lower than found in a 2016 Cochrane review (5–18%)[21]. The fact that there was no significant difference in recurrence between groups 1 and 2 in our study leads us to conclude that anatomic results of SLF are sustainable. Despite several patients lost to follow up and non-responders, a time of observation reaching as high as 11 years made the strength of the study.

In our study and in the literature, the most common site of recurrence was the anterior vaginal wall. For Clark et al*.* [22] recurrence generally occurs in a site different from the one that has been treated. Therefore, the anterior vaginal wall was the preferential site of recurrences when treating the posterior wall and vice versa. For Weemhoff et al*.* [23], there is a greater risk of recurrence when associating SLF and anterior colporrhaphy. Their first hypothesis was a modified distribution of intra-abdominal pressure with a recurrence occurring in the weaker compartment. The second one was that the initial clinical assessment is underestimated in a compartment because the prolapse is predominant in another. When the mainly involved compartment is treated, the other is uncovered. Recent studies [24] including the OPTIMAL [10] and OPUS [25] study found a better anatomic result in the anterior compartment when performing anterior compartment refection with colposuspension in a stage 3 or 4 prolapse. Their recurrence rate and anterior compartment recurrence rate were respectively 25% and 19%. Shkarupa et al. [26] performed a concomitant prosthetic cystocele cure with SLF to prevent cystocele recurrence. This option is questionable considering the controversy surrounding the use of prosthetic material for prolapse treatment, let alone prevention. A recent Cochrane review recommended performing surgical cystocele cures without using prosthetic material because of the high risk of material exposure [27]. This issue therefore remains unanswered and a randomized prospective study evaluating the use of a concomitant cystocele cure is indicated.

Compared to abdominal sacral colpopexy, SLF is associated with shorter hospitalisation stay, faster return to activities of daily living and a smaller cost [28]. Short-term success rates are similar between these two techniques. A Cochrane review [29] noticed a lower rate of recurrent vault prolapse after abdominal sacral colpopexy compared to SLF. However, this review could not find any difference between these two techniques when considering subjective outcomes such as patient satisfaction and the number of women reporting prolapse symptoms. Nowadays SLF is still considered an effective and less morbid surgical technique for pelvic organ prolapse.

Quality of life was high in our population and means of PDFI-20 and PFIQ-7 were satisfactory. Only four patients (7%) had symptomatic prolapse symptoms defined by a PFDI-20 score above 62. Three patients (5%) reported a negative impact on their quality of life in relation with the prolapse. There was no significant difference for the two scores (PFDI-20 and PFIQ-7) between patients with and without recurrence.

The PFDI-20 score inconsistently matches the anatomic result [30]. We can therefore conclude that symptom severity does not reflect the prolapse stage: there remain individual variations. In a prospective study setting, a double examination by two physicians for POP-Q staging could help mitigate the impact of such individual variations.

## Conclusion

This study showed sustainable anatomic and functional results of SLF at medium and long-term. We observed similar anatomic results in groups 1 and 2 with low morbidity. Good quality of life was reported in a great majority of, independent of the presence of a recurrence.

In case of recurrence, it occurred almost exclusively in the anterior compartment as previously reported in the literature. Further research in this field is required to explore anterior recurrence in SLF. A randomised study assessing the role of a concomitant cystocele cure when performing SLF as means of prevention for anterior compartment recurrence is required.

## Data Availability

The datasets used during the current study are available from the corresponding author upon reasonable request.

## Abbreviations

CRADI: Colorectal-Anal Distress Inventory
PFDI-20: Pelvic floor distress inventory
PFIQ-7: Pelvic Floor Impact Questionnaire
POPDI: Pelvic Organ Prolapse Distress Inventory
SLF: Sacrospinous ligament fixation
UDI: Urinary Distress Inventory
